# Response of Canola productivity to integration between mineral nitrogen with yeast extract under poor fertility sandy soil condition

**DOI:** 10.1038/s41598-022-24645-0

**Published:** 2022-11-23

**Authors:** Mohamed Ebaid, M. A. Abd El-Hady, M. E. El-Temsah, Y. A. El-Gabry, Y. M. Abd-Elkrem, H. Hussein, M. A. Abdelkader, T. A. Eliwa, Eslam Salama, Ahmed M. Saad

**Affiliations:** 1 Plant Production Department, Arid Lands Cultivation Research Institute (ALCRI), City of Scientific Research and Technological Applications (SRTA-City), New Borg El-Arab City, Alexandria 21934 Egypt; 2 Agronomy Department, Faculty of Agriculture, Ain Shams University, Cairo, 11566 Egypt; 3 Field Crops Research Department, National Research Center, Dokki, Giza, 12622 Egypt; 4 Environment and Natural Materials Research Institute (ENMRI), City of Scientific Research and Technological Applications (SRTA-City), New Borg El-Arab City, Alexandria 21934 Egypt; 5 Agronomy Department, Faculty of Agriculture, Benha University, Benha, 13511 Egypt

**Keywords:** Ecology, Plant sciences

## Abstract

Canola is one of the important oil crops and is considered the most promising oil source and adapts to reclaimed soil conditions. The current study aimed to evaluate the influence of yeast extract (YE) integrated with nitrogen (N) rates and treatments were arranged as follows: Control (without F0), 95 kg N ha^−1^ (F1), 120 kg N ha^−1^ (F2), 142 kg N ha^−1^ (F3), 95 kg N ha^−1^ + YE (F4), 120 kg N ha^−1^ + YE (F5) and 142 kg N ha^−1^ + YE (F6) on physico-chemical properties, yield and its components for three Canola genotypes i.e. AD201 (G1), Topaz and SemuDNK 234/84 under the sandy soil. In this work, Results reveal that increasing rates of Nitrogen fertilization from 95 kg N ha^−1^ to 142 kg N ha^−1^ have a great effect on physicochemical properties yield and its components. The result proved that 142 kg N ha^−1^ with yeast treatment was the best treatment for three Canola genotypes. Also, the result showed that seed yield was positively correlated with Chl. a/b ratio, plant height, number of branches/plant, number of pods/plant, and number of seeds/pod, and a strong negative correlation was detected between seed oil percentage when the amount of nitrogen fertilization applied without or with yeast extract is increased.

## Introduction

Egypt is suffering from a great shortage of edible oils, wherein the gap between the total local production and imported oil is about 92%^1^. In Egypt, the cultivated area of oil crops is relatively little due to the excessive competition between them and other strategic winter season crops on the limited arable land in the Nile valley and Delta. Cultivation of oil crops such as Canola (*Brassica napus* L.) may supply a chance to overcome of deficiency of edible oil production in Egypt, because it can cultivate in various regions in comparison with the other oil crops, due to its ability to tolerate abiotic environmental stress such as salinity, drought, etc^2^. Canola is considered as a promising crop for crude oil production in many countries (14.7% of total edible oil in the world) because it contains high percentage and the good quality oil, wherein oil has a high content of omega 3, vitamin E, lowest saturated fatty acids, erucic acid, and glucosinolates^3^. Thus, it is recognized as efficient food by medicine^4^. Furthermore, in industry, oil is used to produce detergents, varnish, cleaning products, leather, rubber component, and biodiesel (rape methyl ester)^5^, the residual mass after oil extraction is rich in proteins and can be used for animal feeding^6^.

Chemical fertilizers are the main source of nitrogen (N) input in crop production systems. N plays a critical role in agriculture by increasing crop yield, and it is considered an essential element and occupies a noticeable place in the plant metabolism system; all vital processes in the plant are correlated with protein in which N is a fundamental constituent^7^. N enhances photosynthetic processes, leaf area production, leaf area duration, and net assimilation rate; consequently, increasing the production of the yield^8^. Fertilization management practices are one of the mainly important agro-management factors that affect the yield and its components of crops, in particular those grown in the newly reclaimed desert soils^9^. The optimizing application of nitrogen fertilization rates leads to improve characteristics of the Canola crop, wherein there is positively correlated with soil N level and Canola traits i.e. plant height, number of branches/plant, number of pods/plant, seed yield and oil yield^10^, yield traits are affected directly by N as a result of increased stem length, a higher number of flowering branches, total plant weight, seeds per pod, number and weight of pods and seeds/pod^11^. The chemical nitrogen fertilizers, on another hand, have numerous disadvantages such as insufficient supply or adulteration or unavailability of fertilizer at the time of applied. However, excessive N fertilizer use that goes beyond what crops actually need has had unfavorable effects on the quality of the soil, water, and air. Among these include soil acidification, nitrogen leaking into groundwater, and nitrous oxide (N_2_O) emissions, a strong greenhouse gas that accelerates global warming^12^. Furthermore, continuous application of chemical fertilizers creates soil contamination effect on the environment and it consumes great energy and cost during the chemical production process^13^. So, integration between chemical and bio-fertilization is a successful key for these problems. The combination between bio-fertilization and chemical sources of nitrogen provides crops with nitrogen requirements and decreased the pH, which led to enhance the availability of trace elements that improve plant growth^14^, bio-fertilizers to be a safe alternative to chemical fertilizers to minimize the ecological disturbance, capital and energy of chemical industry process^13^.

Dry bread yeast (*Saccharomyces cerevisiae*) is a kind of bio-fertilizers applied to soil or foliar spray for fertilizing the crops^15^. Dry bread yeast plays a valuable role in vegetative and reproductive growth stages, wherein it contains many nutrients capable to produce growth regulator hormones such as auxins and gibberellins, and it improves the simulative growth compounds that act to enhance the process of photosynthesis^16^, cell division and growth of the plant^17^, improve flower formation, enhance nucleic acid protein and accumulation of carbohydrates^18^. Spraying potato plants in five concentrations of dry bread yeast caused a significant increase in plant height, a number of branches, shoot dry matter, the number of tubers/plant, the rate of tuber weight, and plant yield^19^. Used dry bread yeast has improved growth and productivity in some vegetable crops^19^. Yeast has an opportunity to generate a band of enzymes that transform sugars into alcohol and CO_2_, which is utilized by plants in the photosynthetic process and leads to many plant hormones such as cytokinins, gibberellins, and auxins additionally, vitamins like B1, B2, B6 and B12 similarly, dry yeast possess a stimulatory influence on cell division and expansion, protein, nucleic acid synthesis, and chlorophyll formation. Since yeast is a natural source of cytokinins and protein, the function of yeast extract in enhancing cell division and enlargement of the cell^18^ so the yeast extract may be responsible for the increase in canola growth and productivity. Therefore, the present study was planned to improve and maximize the productivity of Canola under sandy soil conditions (Nobaria, Behaira Governorate, Egypt) by studying the effect of integration between different rates of nitrogen fertilization and dry bread yeast extract (*Saccharomyces cerevisiae*) on Canola. Photosynthetic pigments, growth, yield traits, and physico-chemical properties of the oil.

## Results and discussion

### Photosynthetic pigments

Based on the analysis of variance, data of Photosynthetic pigments as presented in Table 1 indicate that photosynthetic pigments as chlorophyll a (Chl. a) had non-significant for three Canola genotypes AD201 (G1), Topaz (G2) and SemuDNK 234/84 (G3), but chlorophyll b (Chl. b) and chlorophyll a/b ratio (Chl. a/b) had significant difference for three genotypes. Chl. a, Chl. b and Chl. a/b were positively responded to different N application i.e. without nitrogen fertilization (control F0), 95 kg N ha^−1^ (F1), 120 kg N ha^−1^ (F2) and 142 kg N ha^−1^ (F3) (without yeast); and integrated between nitrogen fertilization and yeast extract (YE) treatments as follows: 95 kg N ha^−1^ + YE (F4), 120 kg N ha^−1^ + YE (F5) and 142 kg N ha^−1^ (F6) (with yeast), data indicated that F5 and F6 gave the highest values of Chl. a and Chl. a/b ratio and lowest values of Chl. b Table 1. Interaction data showed that three Canola genotypes that were fertilized with N without yeast or with yeast had a slight difference with statistically significant in chl. a. The highest values of Chl. an obtained by G2 under F5 treatment followed by G1 under F6 treatments. In respect to Chl. a/b ratio, statistical analysis showed that Interaction between Canola genotypes treated with N applications without or with yeast had a significant difference whereas the highest values were recorded when Canola genotypes G3 and G2 fertilized with F6 and F5 with slight differences. While the interaction was significant between N treatments and Canola genotypes for Chl. b. and Canola genotype (G1) gave the highest value when treated with F1. Generally, F6 and F5 improve the contents of chl. a and chl. a/b ratio for three Canola genotypes Table 1. Chl. contents were increased in plants grown under middle and high N conditions as compared with plants grown under low N conditions, which significantly affected photochemical processes^20^. N is a fundamental element for leaf plants, insufficient N supply lead to decreased photosynthetic rate in plants^21^, this occurs to many factors such as a decrease in pigment degradation^22^, reduction in stomatal conductance^23^ and a decline in the light and dark reaction of photosynthesis. Canola is a nitrophilous plant, wherein a high concentration of NO_3_ in the culture media results in higher Chl. contents in the plant leave compared with controls^20^. The Chl. a/b ratio can be a valuable indicator of N element within a leaf because this ratio must be positively related to the ratio of PSII cores to light-harvesting chlorophyll-protein complex (LHCII)^24^. LHCII contains the majority of Chl. b, consequently it has a lower Chl a/b ratio than other Chl. binding proteins associated with PSII^25^. Thus, Chl. a/b ratios should increase with decreasing N availability, especially under high light conditions^26^, the Chl. a/b ratio and the ratio of PSII to Chl. are independent of N availability for spinach^27^, and lower Chl. a/b ratios were noticed when plants were subjected to low N^28^, while Kitajima and Hogan^29^ revealed that the Chl. a/b ratio increased when Chl. content decreased in response to N restriction in photosynthetic cotyledons in leaves of seedlings of four tropical woody species in the Bignoniaceae, and Bungard et al.^30^ demonstrated that there is a tiny response in Chl. a/b ratios to light or N. The yeast includes bio-regulators i.e. plant growth regulators and endogenous plant hormones, which enhance photosynthesis, also it produces 5-Aminolevulinic acid which is vital to tetrapyrrole biosynthesis and biochemical processes in plants, including heme and Chl. biosynthesis^25^.

**Table 1:** Photosynthetic pigments for the three Canola genotypes under different N applications without and with yeast extract.

Studied factor		Chlorophyll a	Chlorophyll b	Chl. a/b ratio
**Genotypes (G)**				
G1 (AD201)		4.06 ± 0.132a	1.56 ± 0.046a	2.68 ± 0.157b
G2 (Topaz)		4.11 ± 0.117a	1.47 ± 0.048b	2.87 ± 0.167ab
G3 (SemuDNK 234/84)		4.16 ± 0.092a	1.44 ± 0.058c	3.00 ± 0.177a
**Fertilizer (F)**				
F0 (control)		2.96 ± 1.657e	1.56 ± 0.026d	1.91 ± 0.087d
F1 (95 kg N ha^−1^ without yeast)		3.48 ± 0.123d	1.73 ± 0.021a	2.02 ± 0.084d
F2 (120 kg N ha^−1^ without yeast)		4.35 ± 0.046bc	1.66 ± 0.015b	2.63 ± 0.049c
F3 (142 kg N ha^−1^ without yeast)		4.25 ± 0.068c	1.58 ± 0.020c	2.70 ± 0.062c
F4 (95 kg N ha^−1^ with yeast)		4.42 ± 0.054bc	1.45 ± 0.060e	3.07 ± 0.115b
F5 (120 kg N ha^−1^ with yeast)		4.70 ± 0.035a	1.23 ± 0.021f	3.84 ± 0.066a
F6 (142 kg N ha^−1^ with yeast)		4.62 ± 0.150ab	1.22 ± 0.025f	3.80 ± 0.135a
**Interaction**				
G1	F0	2.68 ± 0.009h	1.63 ± 0.005de	1.64 ± 0.027h
	F1	3.16 ± 0.009g	1.81 ± 0.003a	1.74 ± 0.007gh
	F2	4.20 ± 0.012de	1.71 ± 0.012b	2.45 ± 0.023de
	F3	4.34 ± 0.010bcde	1.51 ± 0.009h	2.88 ± 0.011c
	F4	4.59 ± 0.009abcd	1.61 ± 0.012ef	2.85 ± 0.017c
	F5	4.69 ± 0.035abc	1.31 ± 0.009i	3.59 ± 0.009b
	F6	4.76 ± 0.018 ab	1.32 ± 0.008i	3.61 ± 0.025b
G2	F0	3.04 ± 0.025gh	1.51 ± 0.010h	2.02 ± 0.009fg
	F1	3.58 ± 0.021f	1.67 ± 0.009c	2.14 ± 0.006f
	F2	4.52 ± 0.003abcde	1.62 ± 0.006de	2.79 ± 0.009c
	F3	4.12 ± 0.195e	1.59 ± 0.009f	2.59 ± 0.13cd
	F4	4.35 ± 0.006bcde	1.52 ± 0.003gh	2.86 ± 0.009c
	F5	4.81 ± 0.007a	1.21 ± 0.003j	3.97 ± 0.015a
	F6	4.37 ± 0.472bcde	1.17 ± 0.003k	3.75 ± 0.410ab
G3	F0	3.15 ± 0.312g	1.54 ± 0.009g	2.05 ± 0.184fg
	F1	3.71 ± 0.307f	1.71 ± 0.009b	2.17 ± 0.170ef
	F2	4.33 ± 0.027bcde	1.64 ± 0.007d	2.64 ± 0.026cd
	F3	4.30 ± 0.062cde	1.64 ± 0.006d	2.62 ± 0.045cd
	F4	4.31 ± 0.110cde	1.22 ± 0.006j	3.53 ± 0.076b
	F5	4.62 ± 0.061abcd	1.16 ± 0.014k	3.97 ± 0.061a
	F6	4.73 ± 0.006abc	1.17 ± 0.006k	4.04 ± 0.023a
**ANOVA**	**df**			
Genotypes (G)	2	< 0.001	< 0.001	< 0.001
Fertilizer (F) level	6	< 0.001	< 0.001	< 0.001
G × F	12	< 0.001	< 0.001	< 0.001

### Yield and its attributes

Comparing of mean data through the Duncan Multiple Range Test in the probability level of 5%, data showed significant differences among the Canola genotypes for the highest plant (cm), branches number/plant, and pods number/plant. On contrary, there wasn’t a significant difference for seed number/pods, seed yield (t ha^−1^), biological yield (t ha^−1^), and harvest index, wherein G2 gave the highest value for the highest plant (cm). In the same trend, G2 gave the highest values of branches No./plant and pods No./plant followed by G3 for the previous two treats Table 2. All examined N without or with yeast caused a significant difference in yield and its attributes, wherein F6 positively affected on abovementioned traits and gave the highest values on the highest plant (cm), branches No./plant, pods No./plant, seed No./pods, seed yield (t ha^−1^), and harvest index. While the highest values of biological yield (t ha^−1^) were obtained with F3, F6, and F5, respectively Table 2.

**Table 2:** Growth, yield and its attributes for the three Canola genotypes under different N applications without and with yeast extract.

Studied factor		Plant height (cm)	Number of branches/plant	Pods number/plant	Number of seed/pods	Seed yield (t ha^−1^)	Biological yield (t ha^−1^)	Harvest index
**Genotypes (G)**								
G1 (AD201)		155.74 ± 3.394b	4.86 ± 0.389b	148.68 ± 6.362b	21.34 ± 1.094a	2.25 ± 0.138a	7.71 ± 0.323a	0.28 ± 0.010a
G2 (Topaz)		161.33 ± 3.886a	7.48 ± 0.510a	158.89 ± 6.870a	22.40 ± 1.15a	2.38 ± 0.151a	7.77 ± 0.289a	0.30 ± 0.011a
G3 (SemuDNK 234/84)		151.69 ± 2.442b	7.05 ± 0.616a	155.29 ± 6.761a	21.90 ± 1.308a	2.35 ± 0.174a	7.60 ± 0.400a	0.30 ± 0.014a
**Fertilizer (F)**								
F0 (control)		75.88 ± 2.263 g	2.78 ± 0.57e	76.87 ± 1.56f.	9.78 ± 0.670f.	0.73 ± 0.138e	3.18 ± 0.359c	0.23 ± 0.009c
F1 (95 kg N ha^−1^ without yeast)		151.78 ± 1.077f.	4.44 ± 0.338d	128.11 ± 1.327e	17.78 ± 0.521e	1.84 ± 0.126d	7.28 ± 0.394b	0.25 ± 0.009c
F2 (120 kg N ha^−1^ without yeast)		164.33 ± 2.261 d	6.22 ± 0.465c	153.44 ± 1.834c	20.89 ± 0.309 d	2.26 ± 0.084c	7.80 ± 0.284b	0.29 ± 0.006b
F3 (142 kg N ha^−1^ without yeast)		182.89 ± 2.988 b	7.56 ± 0.294b	184.78 ± 1.544 b	27.56 ± 0.648b	2.99 ± 0.076 b	9.61 ± 0.180 a	0.31 ± 0.009b
F4 (95 kg N ha^−1^ with yeast)		159.00 ± 1.080 e	5.44 ± 0.377c	145.67 ± 1.624d	20.00 ± 0.500d	2.11 ± 0.077c	7.36 ± 0.322b	0.29 ± 0.007b
F5 (120 kg N ha^−1^ with yeast)		170.89 ± 2.282 c	8.33 ± 0.553b	182.56 ± 3.520b	25.89 ± 0.512c	2.76 ± 0.071b	9.03 ± 0.493 a	0.31 ± 0.015b
F6 (142 kg N ha^−1^ with yeast)		189.00 ± 2.449a	10.44 ± 0.766a	208.56 ± 2.410a	31.22 ± 0.494a	3.59 ± 0.090a	9.60 ± 0.351a	0.38 ± 0.016a
**Interaction**								
G1	F0	74.17 ± 2.568i	1.33 ± 0.425j	74.40 ± 3.482n	9.35 ± 0.627i	0.66 ± 0.147i	3.04 ± 0.714g	0.22 ± 0.008i
	F1	148.33 ± 1.453k	3.33 ± 0.333i	124.00 ± 2.082l	17.00 ± 0.577h	1.66 ± 0.110h	6.94 ± 0.615ef	0.24 ± 0.006ghi
	F2	168.00 ± 2.082f	4.67 ± 0.333gh	147.67 ± 1.764i	21.33 ± 0.333f	2.52 ± 0.060ef	8.54 ± 0.511bcd	0.30 ± 0.012cdefg
	F3	181.67 ± 1.333 d	6.67 ± 0.333de	180.33 ± 2.848e	26.67 ± 0.333cd	2.84 ± 0.070de	9.94 ± 0.340a	0.29 ± 0.014cdef
	F4	156.33 ± 2.333 hi	4.00 ± 0.123hi	141.00 ± 3.464j	20.00 ± 0.577fg	2.11 ± 0.130g	7.68 ± 0.581def	0.28 ± 0.009defgh
	F5	174.00 ± 3.512 e	6.33 ± 0.333ef	172.33 ± 2.404f	24.67 ± 0.333e	2.52 ± 0.073ef	8.15 ± 0.743 cde	0.31 ± 0.020 cdef
	F6	187.67 ± 0.882c	7.67 ± 0.333cd	201.00 ± 2.517c	30.33 ± 1.202 b	3.40 ± 0.196b	9.69 ± 0.577 ab	0.35 ± 0.030abc
G2	F0	77.00 ± 0.789i	3.67 ± 0.457hi	79.20 ± 0.759m	10.08 ± 0.472i	0.76 ± 0.092i	3.33 ± 0.625g	0.23 ± 0.016hi
	F1	154.00 ± 1.527ij	5.33 ± 0.333fg	132.00 ± 0.577k	18.33 ± 0.333h	1.90 ± 0.080gh	7.61 ± 0.543def	0.25 ± 0.027ghi
	F2	169.33 ± 0.333f	7.67 ± 0.333cd	159.00 ± 2.082g	21.00 ± 0.5772f	2.14 ± 0.092g	7.38 ± 0.315 def	0.29 ± 0.002 defgh
	F3	193.67 ± 0.882b	8.33 ± 0.333bc	188.6 ± 1.453 d	29.67 ± 0.882b	3.25 ± 0.097bc	9.65 ± 0.273ab	0.34 ± 0.009bcd
	F4	162.00 ± 0.577g	6.33 ± 0.333ef	149.33 ± 0.882 i	21.33 ± 0.882f.	2.25 ± 0.175fg	7.60 ± 0.684 def	0.30 ± 0.003cdefg
	F5	175.33 ± 2.603e	9.33 ± 0.333b	188.67 ± 5.897d	25.33 ± 0.333de	2.80 ± 0.077de	9.45 ± 0.107ab	0.30 ± 0.009 cdefg
	F6	198.00 ± 0.577a	11.67 ± 0.333a	215.33 ± 2.603a	31.00 ± 0.577ab	3.57 ± 0.098ab	9.37 ± 0.636ab	0.38 ± 0.018ab
G3	F0	76.50 ± 1.333i	3.33 ± 0.627i	77.00 ± 2.378mn	9.90 ± 1.025i	0.78 ± 0.056i	3.18 ± 0.256g	0.24 ± 0.028ghi
	F1	153.0 ± 0.577j	4.67 ± 0.333gh	128.33 ± 0.667k	18.00 ± 1.527h	1.95 ± 0.387gh	7.28 ± 1.037ef	0.26 ± 0.014fghi
	F2	155.67 ± 0.333hij	6.33 ± 0.333ef	153.67 ± 0.882h	20.33 ± 0.667f	2.11 ± 0.143g	7.48 ± 0.443def	0.28 ± 0.018defgh
	F3	173.33 ± 0.333e	7.67 ± 0.333cd	185.33 ± 0.882d	26.33 ± 0.882cd	2.89 ± 0.078d	9.23 ± 0.265 abc	0.31 ± 0.012cdef
	F4	158.67 ± 0.333h	6.00 ± 0.233ef	146.67 ± 0.882 i	18.67 ± 0.333gh	1.96 ± 0.044gh	6.81 ± 0.455f	0.29 ± 0.021defgh
	F5	163.33 ± 0.333g	9.33 ± 0.667b	186.6 ± 5.364d	27.67 ± 0.667c	2.95 ± 0.042cd	9.51 ± 1.328ab	0.32 ± 0.047cde
	F6	181.33 ± 0.333d	12.00 ± 0.567a	209.33 ± 2.156b	32.33 ± 0.333a	3.80 ± 0.097a	9.73 ± 0.839ab	0.40 ± 0.039a
**ANOVA**	**df**							
Genotypes (G)	2	< 0.001					< 0.001	< 0.001
Fertilizer (F) level	6	< 0.001					< 0.001	< 0.001
G × F	12	< 0.001					< 0.001	< 0.001

The interaction between the Canola genotype and different N rates without or with yeast extract as shown in Table 2, demonstrated a significant difference. Data showed that the highest values of plant height and pods No./plant were recorded by G2 under F6 and the highest values of branches No./plant, seed No./pods, and seed yield (t ha^−1^) got by G3 and G2 under F6. There was a slight difference with statistically significant biological yield (t ha^−1^) and highest values established by G1 under F3 and F6; and G2 and G3 under F3, F5, and F6 respectively; and the highest values of harvest index recorded by G1, G2 and G3. under F6. Generally, data proved that 142 kg N/ h^−1^ + YE (F6) was enhanced the yield and its components of three Canola genotypes i.e. AD201 (G1), Topaz (G2), and SemuDNK 234/84 (G3). Many researchers reported that there are significant differences among Canola varieties and growth and yield traits are significantly increased by increasing N rates^11^. Increasing N fertilizer rates significantly increased most of the yield and its components^31^, N enhances metabolites synthesized by the plant which leads to more transformation of photosynthesis to reproductive parts, and induces different physiological mechanisms to access the nutrient^32^. Yeast extract as bio-fertilizer had a significant and positive effect on plant height and yield traits of Canola. The role of bread yeast in increasing the growth and yield traits; may be due to the content of yeast to many important nutrients elements i.e. N, Mg, Ca, Zn, Cu, and Fe, and the production of some growth regulators such as Auxin and Gibberellin and cytokinin which is necessary for plant biological processers especially photosynthesis and cell division and elongation^33^. Also, Yeast extract had stimulatory effects on cell division and enlargement, protein and nucleic acid synthesis, and chlorophyll formation^34^, in addition to its content of cryoprotective agent, i.e. sugars, protein, amino acids, and also several vitamins^35^. Consequently, it improves growth, flowering, and fruit set and formation and increases yield^34^.

### Correlation of Canola seed yield and chlorophyll a/b ratio

**Figure 1:**
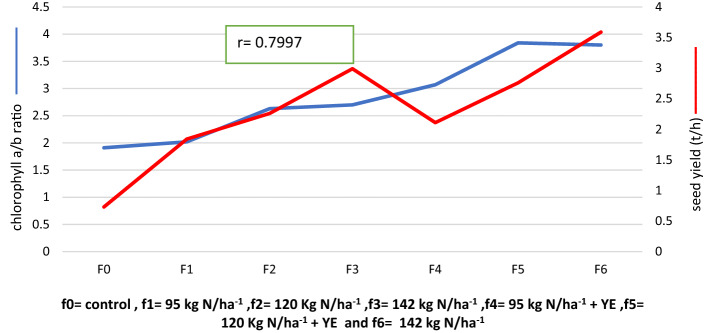
Correlation of Canola seed yield (t/h) and chlorophyll a/b ratio as affected by different nitrogen rates without and with yeast extract.

Partial correlation coefficients of Canola seed yield and Chl. a/b ratio is given in Fig. 1. This result showed that seed yield was positively correlated with Chl. a/b ratio when the amount of N applied without or with yeast extract is increased. Chl. a/b ratio can be an important indicator of N within a leaf, this ratio must be positively related to photosynthesis and biological processers which reflect on seed yield.

### Correlation of Canola seed yield and its attributes

**Figure 2:**
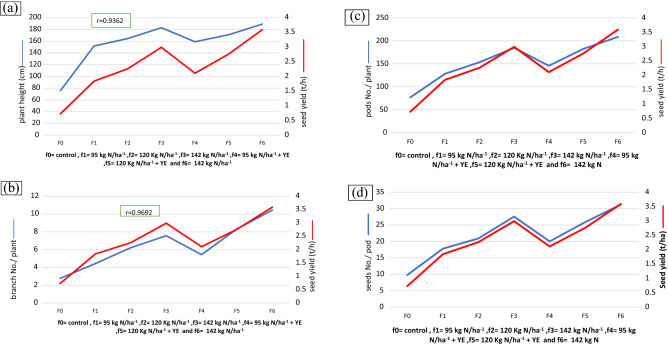
(**a**) Correlation of Canola seed yield (t/h) and plant height (cm) as affected by different nitrogen rates without and with yeast extract, (**b**) Correlation of Canola seed yield (t/h) and branch No/plant as affected by different nitrogen rates without and with yeast extract, (**c**) Correlation of Canola seed yield (t/h) and pods No/ plant as affected by different nitrogen rates without and with yeast extract, and (**d**) Correlation of Canola seed yield (t/h) and seeds No/ pod as affected by different nitrogen rates without and with yeast extract.

Correlations of seed yield and yield components of Canola are a function of the plant height, number of branches/plant, number of pods/plant, and number of seeds/pod as shown in Fig. 2a–d. These results proved that grain yield was strongly positively correlated with some of the abovementioned traits when N fertilization increased without or with yeast extract. Sufficient N contributes to enhance physiological processes, improves growth, flowering, seed formation, and the seed yield finally.

### Chemical properties

Regarding results of the oil yield (t ha^−1^), seed oil %, protein %, N % in seed, and N% in straw as presented in Table 3, data showed significant differences among three Canola genotypes; AD201 (G1), Topaz (G2) and SemuDNK 234/84 (G3), excepted oil yield had non-significant difference. G1 was surpassed in oil %; G2, G3 surpassed in protein % and N % in seed, and G3 surpassed in N% in straw. Different N fertilization applies without or with yeast extract had a significant effect on the abovementioned traits, wherein F6 treatment gave the highest oil yield, protein %, N % in seed, and N% in straw, while seed oil % significantly increased with F1 and F4 treatments. There was significant interaction concerning with abovementioned traits, Table 3, as well as the highest values of seed oil yield (t ha^−1^), protein % in seeds, and nitrogen % in seeds were obtained with G1, G2, and G3 when treated with F6. Wherein the highest values of oil % were obtained by G1 under F1 and F4 treatments. Concerning N% in straw was increased by increasing the rate of N fertilizer application and the highest value was recorded by adding F6 to G3^36^. Seed oil percentage was decreased by increasing nitrogen rates; the effect of interaction between Canola cultivars and nitrogen fertilization treatments was significant on seed oil. % High rates of N led to decreases in seed oil % and increase in protein concentrations in Canola seed^37^, the increase in seed protein % because N is an integral part of protein and the protein of Canola.

**Table 3:** Effect of different N applications without and with yeast extract on oil yield, oil %, protein %, N % in seed and N% in straw for the three Canola genotypes.

Studied factor		Oil yield (t/ha)		Seed oil %	Protein %	N % in seed	N % in straw
**Genotypes (G)**							
G1 (AD201)		938.99 ± 73.7a		41.97 ± 0.408a	18.98 ± 0.811b	3.04 ± 0.130b	0.57 ± 0.025c
G2 (Topaz)		982.88 ± 78.7a		41.28 ± 0.335b	19.70 ± 0.797a	3.15 ± 0.127a	0.61 ± 0.023b
G3 (SemuDNK 234/84)		959.14 ± 78.9a		41.15 ± 0.488b	20.00 ± 0.750a	3.20 ± 0.120a	0.67 ± 0.024a
**Fertilizer (F)**							
F0 (control)		289.80 ± 19.3e		39.52 ± 0.233e	12.62 ± 0.159g	2.02 ± 0.025g	0.42 ± 0.014g
F1 (95 kg N ha^−1^ without yeast)		805.00 ± 53.6d		43.91 ± 0.259a	18.02 ± 0.227f	2.88 ± 0.036f	0.55 ± 0.019f
F2 (120 kg N ha^−1^ without yeast)		956.00 ± 36.3c		42.33 ± 0.248 b	18.64 ± 0.308e	2.98 ± 0.049e	0.59 ± 0.014e
F3 (142 kg N ha^−1^ without yeast)		1210.00 ± 32.8b		40.46 ± 0.232 d	22.81 ± 0.195b	3.65 ± 0.031b	0.63 ± 0.017c
F4 (95 kg N ha^−1^ with yeast)		918.00 ± 31.8c		43.59 ± 0.157 a	19.49 ± 0.229d	3.12 ± 0.036d	0.61 ± 0.017d
F5 (120 kg N ha^−1^ with yeast)		1136.00 ± 23.2b		41.22 ± 0.327 c	21.28 ± 0.195c	3.40 ± 0.031c	0.72 ± 0.020b
F6 (142 kg N ha^−1^ with yeast)		1407.00 ± 35.8a		39.22 ± 0.346 e	24.06 ± 0.194a	3.85 ± 0.031a	0.76 ± 0.017a
**Interaction**							
G1	F0	267 ± 18.6g		40.26 ± 0.159fg	12.13 ± 0.207i	1.94 ± 0.0.033i	0.37 ± 0.011n
	F1	743 ± 51.6f		44.73 ± 0.176a	17.33 ± 0.296g	2.77 ± 0.047g	0.49 ± 0.015l
	F2	1068 ± 21.6cd		42.40 ± 0.173cd	17.67 ± 0.233g	2.83 ± 0.037g	0.56 ± 0.027jk
	F3	1161 ± 29.5c		40.87 ± 0.088ef	22.47 ± 0.549b	3.60 ± 0.088b	0.59 ± 0.017i
	F4	929 ± 58.2de		43.87 ± 0.088ab	18.67 ± 0.145f	2.99 ± 0.023f	0.57 ± 0.024jk
	F5	1067 ± 39.7cd		42.33 ± 0.388cd	20.70 ± 0.306d	3.31 ± 0.049d	0.68 ± 0.035ef
	F6	1336 ± 78.2ab		39.30 ± 0.208hi	23.87 ± 0.521a	3.82 ± 0.083a	0.72 ± 0.020d
G2	F0	297 ± 12.6g		39.09 ± 0.234hi	12.62 ± 0.130hi	2.02 ± 0.021hi	0.42 ± 0.009m
	F1	825 ± 35.4ef		43.43 ± 0.260b	18.03 ± 0.186fg	2.89 ± 0.030fg	0.55 ± 0.012k
	F2	889 ± 41.6ef		41.53 ± 0.273de	18.77 ± 0.240f	3.00 ± 0.038f	0.58 ± 0.015ij
	F3	1327 ± 34.9ab		40.87 ± 0.291ef	23.07 ± 0.219b	3.69 ± 0.035b	0.62 ± 0.012gh
	F4	969 ± 69.4de		43.10 ± 0.289bc	19.87 ± 0.318e	3.18 ± 0.051e	0.61 ± 0.020h
	F5	1147 ± 22.4c		40.93 ± 0.333ef	21.37 ± 0.260cd	3.42 ± 0.042cd	0.71 ± 0.023d
	F6	1426 ± 36.1a		40.00 ± 0.153fgh	24.20 ± 0.173a	3.87 ± 0.028a	0.74 ± 0.026c
G3	F0	305 ± 59.8g		39.21 ± 0.397hi	13.09 ± 0.121h	2.09 ± 0.019h	0.47 ± 0.004l
	F1	847 ± 66.1ef		43.57 ± 0.441b	18.70 ± 0.173f	2.99 ± 0.028f	0.61 ± 0.005h
	F2	910 ± 63.4e		43.07 ± 0.203bc	19.50 ± 0.416e	3.12 ± 0.067e	0.64 ± 0.003g
	F3	1144 ± 25.0c		39.63 ± 0.219gh	22.90 ± 0.115b	3.66 ± 0.018b	0.69 ± 0.003e
	F4	857 ± 18.8ef		43.80 ± 0.153b	19.93 ± 0.033e	3.19 ± 0.005e	0.66 ± 0.009f
	F5	1193 ± 20.0bc		40.40 ± 0.252fg	21.77 ± 0.067c	3.48 ± 0.011c	0.78 ± 0.007b
	F6	1458 ± 64.2a		38.37 ± 0.833i	24.10 ± 0.351a	3.86 ± 0.056a	0.82 ± 0.007a
**ANOVA**	**df**						
Genotypes (G)	2	< 0.001					
Fertilizer (F) level	6	< 0.001					
G × F	12	< 0.001					

### Correlation of Canola seed yield and seed oil percentage

**Figure 3:**
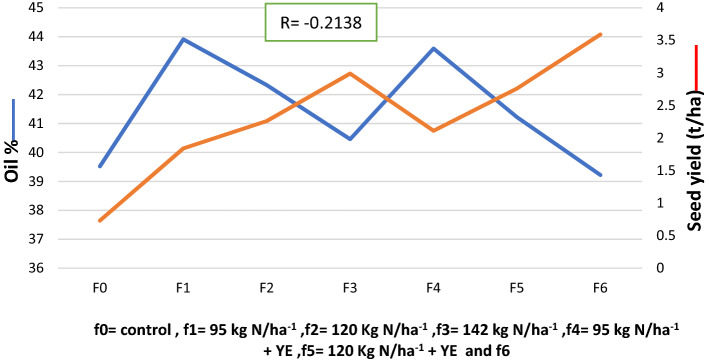
Correlation of Canola seed yield (t h^−1^) and oil % as affected by different nitrogen rates without and with yeast extract.

A strong negative correlation was detected between seed oil percentage as shown in Fig. 3. The result indicates that seed oil percentage decreases with increasing in different N fertilization rates without or with yeast extract. That’s a negative correlation between seed yield and seed oil %; it might be due to N application which results in delaying maturity leading to poor seed filling and a greater proportion of green seed^38^.

### Physico-chemical properties of Canola oil

The effects of different N application rates without or with yeast extract on Canola genotypes on physico-chemical properties i.e. Acid value (mg g^−1^), saponification number (mg g^−1^) and peroxide value (mg kg^−1^) were shown in Table 4. Data of chemical properties of Canola oil showed significant differences among Canola genotypes, the highest acid value and peroxide value were obtained from G2 followed by G1 and G3, respectively, while the highest saponification number was obtained by G3 followed by G1 and G2, respectively.

**Table 4:** Oil properties for three Canola genotypes under different N applications without and with yeast extract.

Studied factor		Acid value (mg g^−1^)		Saponification number (mg g^−1^)	Peroxide value (mg kg^−1^)
**Genotypes (G)**					
G1 (AD201)		1.92 ± 0.111ab		142.76 ± 4.03b	7.90 ± 0.187b
G2 (Topaz)		2.47 ± 0.163a		138.94 ± 4.08c	9.01 ± 0.199a
G3 (SemuDNK 234/84)		1.69 ± 0.104b		144.66 ± 3.75 a	7.83 ± 0.179b
**Fertilizer (F)**					
F0 (control)		2.74 ± 0.237a		177.74 ± .859a	9.65 ± 0.242a
F1 (95 kg N ha^−1^ without yeast)		2.49 ± 0.215b		154.56 ± 0.747b	8.94 ± 0.224b
F2 (120 kg N ha^−1^ without yeast)		2.02 ± 0.171c		142.44 ± 0.765c	8.61 ± 0.197c
F3 (142 kg N ha^−1^ without yeast)		1.68 ± 0.143d		134.00 ± 1.900e	7.97 ± 0.142d
F4 (95 kg N ha^−1^ with yeast)		2.14 ± 0.211c		137.44 ± 1.454d	7.85 ± 0.229d
F5 (120 kg N ha^−1^ with yeast)		1.71 ± 0.116d		126.89 ± 1.135e	7.66 ± 0.167e
F6 (142 kg N ha^−1^ with yeast)		1.40 ± 0.112e		121.78 ± 0.846g	7.03 ± 0.216f
**Interaction**					
G1	F0	2.53 ± 0.33c		179.02 ± 1.533a	9,25 ± 0.259c
	F1	2.30 ± 0.30cde		155.67 ± 1.333c	8.57 ± 0.240de
	F2	2.00 ± 0.21defg		141.33 ± 0.666f	8.23 ± 0.033e
	F3	1.63 ± 0.15ghijkl		135.67 ± 1.333h	7.67 ± 0.088fgh
	F4	1.97 ± 0.18defgh		139.33 ± 0.667g	7.63 ± 0.218fgh
	F5	1.67 ± 0.15ghijkl		127.33 ± 1.667k	7.37 ± 0.120ghi
	F6	1.33 ± 0.19l		121.00 ± 1.533m	6.60 ± 0.115j
G2	F0	3.52 ± 0.06a		175.57 ± 1.333b	10.67 ± 0.047a
	F1	3.20 ± 0.06ab		152.67 ± 1.667d	9.78 ± 0.044b
	F2	2.33 ± 0.23cd		141.33 ± 1.000f	9.35 ± 0.180c
	F3	1.93 ± 0.22efghi		127.00 ± 1.001k	8.52 ± 0.072de
	F4	2.87 ± 0.12b		132.00 ± 1.333i	8.68 ± 0.109d
	F5	1.90 ± 0.23fghij		123.67 ± 1.667l	8.28 ± 0.148e
	F6	1.53 ± 0.19jkl		120.33 ± 0.767m	7.85 ± 0.132f
G3	F0	2.16 ± 0.26def		178.63 ± 0.667a	9.14 ± 0.095c
	F1	1.97 ± 0.23defgh		155.33 ± 0.333c	8.47 ± 0.088de
	F2	1.73 ± 0.29ghijk		144.67 ± 0.666e	8.23 ± 0.133e
	F3	1.47 ± 0.35kl		139.33 ± 1.000g	7.73 ± 0.088fg
	F4	1.60 ± 0.25hijkl		141.00 ± 1.333f	7.23 ± 0.088i
	F5	1.57 ± 0.24ijkl		129.67 ± 1.333j	7.33 ± 0.089hi
	F6	1.33 ± 0.26l		124.00 ± 1.000l	6.63 ± 0.145j
**ANOVA**	**df**				
Genotypes (G)	2	< 0.001			
Fertilizer (F) level	6	< 0.001			
G × F	12	< 0.001			

Where; G1 is AD201, G2 is Topaz, G3 is SemuDNK 234/84, F1 is 95 kg N ha^−1^, F2 is 120 kg N ha^−1^, F3 is 142 kg N ha^−1^ (without bio-fertilizer); F4 is 95 kg N h^−1^ + yeast extract (YE), F5 is 120 kg N ha^−1^ + YE and F6 is 142 kg N ha^−1^ + YE (with bio-fertilizer), G × F is interaction Means in each column with at least one similar letters are not significantly different at the 5% probability leGel using Duncan's new multiple range test.

Data had significant differences among different N application rates without or with yeast extract, by increasing the N rated from F0 to F6 caused decreases in Acid value, Saponification number, and peroxide value. Also, data showed a significant interaction between Canola genotypes and different N application rates without or with yeast extract for all abovementioned traits, wherein the highest values of saponification number were obtained by G1 and G3 under F0 treatment. In addition, the highest values of peroxide value and the acid value were obtained by G2 with F0. The acid value is a physicochemical indicator^38^, wherein oils which have higher acid value posse poor quality^39^, on another hand, Low acid value of Canola genotype shows their higher oil quality. The peroxide value varied between 7.1 and 9.06 meq. O_2_/kg indicates that the tested vegetable oils are fresh, and the lowest initial peroxide value is suitable for consumption^40^. High saponification value indicated that Canola oil possesses normal triglycerides and may be useful in the production of liquid soap and shampoo^41^. Saponification number was significantly different among genotypes and a higher nitrogen rate resulted in an increase in the unsaponifiable matter and led to a decrease in oil acid value and saponification value^42^.

### Fatty acids composition percentages in Canola oil

The main values of fatty acids composition percentages in Canola oil were determined and calculated in the second season Table 5. Gas–liquid chromatographic analysis showed that, saturated fatty acids (Palmitic, 16:0, Stearic, 18:0, Arachidic, 20:0, and Behenic, 22:0) represent about 9.1 of the total fatty acids. Palmitic was the dominant acid among the saturated ones. In respect of unsaturated fatty acids i.e., Oleic acid (18:1), Linoleic (18:2), Linolenic (18:3), and Erucic (22:1), they all represent about 90.9% of total fatty acids. Therefore, Oleic acid (18:1) was the major fatty acid in Canola oil (59.43%) followed by Linoleic (20.80%) and Linolenic (9.02%). Erucic acid was less than 2%.

**Table 5:** Saturated and unsaturated fatty acids (%) in seeds of the three Canola genotypes and different N applications without and with yeast extract.

Treatments		Fatty acids (%)							
		Saturated				Unsaturated			
		Palmitic (16:0)	Stearic (18:0)	Arachidic (20:0)	Behenic (22:0)	Oleic (18:1)	Linoleic (18:2)	Linolenic (18:3)	Erucic (22:1)
Garieties	AD 201	4.79	1.54	2.06	1.15	58.36	21.33	9.16	1.61
	Topaz	4.52	1.22	2.16	1.17	59.57	20.35	8.28	1.77
	Semu DNK	4.01	1.40	2.07	1.20	60.36	20.61	8.91	1.45
Fertilizer	0 k,N h^−1^	**3.03**	*2.87*	*4.05*	*2.31*	**57.96**	**18.03**	**7.58**	*2.32*
	95 k,N h^−1^	3.59	2.00	3.18	1.51	59.68	19.92	8.26	1.87
	120 k,N h^−1^	4.03	1.33	2.08	1.19	60.23	20.71	8.94	1.51
	142 k,N h^−1^	4.38	0.86	0.96	0.90	61.18	21.22	9.52	0.99
	95 k,N h^−1^ + yeast	4.11	2.04	3.24	1.53	58.49	20.07	8.40	2.14
	120 k,N h^−1^ + yeast	4.46	1.39	2.13	1.14	59.36	20.87	8.98	1.66
	142 k,N h^−1^ + yeast	4.73	0.88	0.91	0.85	60.44	21.47	9.69	1.03

Significant values are given in bold and italics.

Data in Table 5, showed slight differences in saturated fatty acids between Canola varieties. AD201(G1) variety contained more amount of Palmitic (4.78%) and Stearic (1.52%) acids followed by Topaz (G2) for Palmitic and SemuDNK 234/84 (V3) for Stearic. However, Behenic acid (1.20%) was higher in G3 than G2 (1.17%), while G2 was the highest in Arachidic acid than G3 variety. These results are in line with those obtained by El Habbasha et al.^43^. They reported that AD 201, Silvo, and Topas (G2) were different in their oil contents of saturated and unsaturated fatty acids. Canola varieties were also slightly differed in their content of the unsaturated fatty acids Table 5, G3 variety contained more amounts of Oleic (60.36%) acid followed by the G2 variety. G1 recorded the lowest amount of Oleic acid (58.36%) in comparison with the other two varieties. On the other hand, G1 showed a high increment in Linoleic and Linolenic acids followed by G3 for Linoleic and Linolenic acids. The second oil quality breeding objective is to reduce the percentage of Linolenic acid from the percent 8–10% to less than 3% while maintaining or increasing the level of Linoleic acid^44^. Lower Linolenic acid is desired to improve the storage characteristics of the oil, while higher Linolenic acid content may be nutritionally desirable. Similar observations were reported by Ref.^45^. Topaz variety recorded the highest value for Erucic acid (1.77%) followed by AD201 variety, whereas Semu DNK gave the lowest value (1.45%). The increase in Erucic acid content in the Topaz variety may be due to the decrease in Oleic acid content^46^. Stated that the concentrations of Oleic and Erucic acids were negatively correlated and a high Oleic acid concentration (> 50%) was always associated with a low Erucic acid concentration (< 4%).

All saturated fatty acids were slightly affected by N fertilizer rate Table 5. Palmitic acid showed an increase (4.73%) with 142 kg N ha^−1^ (F6) with yeast followed by 120 kg N ha^−1^ (F5) with yeast (4.46%), while Stearic, Arachidic, and Behenic acids contents were gradually decreased by increasing N levels up to 142 kg N ha^−1^ (F3) without or with yeast. The highest value of these acids was recorded with control (F0). In contrast, increasing the N rate up to high-level F3 or F6 increased the Oleic, Linoleic, and Linolenic acids and decreased the percent of Erucic acid in comparison with the other treatments Table 5. The unsaturated fatty acids recorded a slight increment as Canola plants fertilized with N except, Linolenic acid which gave the highest value (9.69%) under F6 Table 5. Oleic acid increased as the N rate increased up to F3 or F6, (61.18%), while Erucic acids recorded a higher increment with F0 followed by F4. These results are similar to those obtained by El kholy et al. and El-Beltagi et al.^42,47^ The fatty acid composition of Canola oil is mainly under genetic control but can be modified to some extent by N nutrition. Thus, it can be concluded from these observations that the N affected not only the quantity but also the quality of oil, and to obtain higher oil content in seeds and a better fatty acid profile in the oil of Canola varieties, N fertilizer showed to be applied in balanced doses^45^.

### Nitrogen efficiency indexes

Nitrogen uptake can be assessed by nitrogen efficiency indexes such as nitrogen use efficiency (NUE), nitrogen remobilization efficiency (NRE), nitrogen harvest index (NHI), and nitrogen physiological efficiency (NPE) as presented in Table 6. Nitrogen efficiency indexes differed among Canola genotypes, NUE, NHI and NPE differed among G1, G2, and G3 with non-significant, and NRE was higher in G3 and G2 with non-significant. Previous data showed significant differences among different N application without and with yeast extract, F6 showed the highest values of NUE, NRE, and NHI, while F0 showed the highest values of NPE. There was a significant interaction between Canola genotypes and different N application without and with yeast extract. Nitrogen efficiency indexes are important tools to assess the nutritional status of plants; they can be used to evaluate genotypes that make the best use of applied nutrients. It is also possible to improve management techniques to enhance plant production^48^. Based on Nitrogen efficiency indexes results, the application of nitrogenous nutrients can be improved and, resulting in prevent the overuse of fertilizers, decreasing production costs and environmental damages^49,50^.

**Table 6:** Nitrogen efficiency indexes for three Canola genotypes under different N applications without and with yeast extract.

Studied factor		NUE	NRE	NHI	NPE
**Genotypes (G)**					
G1 (AD201)		21.01 ± 0.592a	98.51 ± 4.132b	65.00 ± 2.249a	22.87 ± 0.812a
G2 (Topaz)		22.20 ± 0.684a	106.75 ± 4.469a	65.76 ± 2.457a	22.15 ± 0.717a
G3 (SemuDNK 234/84)		21.79 ± 0.957a	108.49 ± 5.257a	64.30 ± 2.375 a	21.32 ± 0.690a
**Fertilizer (F)**					
F0 (control)		–	–	41.74 ± 1.089e	29.19 ± 0.683a
F1 (95 kg N ha^−1^ without yeast)		19.32 ± 1.332c	87.15 ± 5.630d	63.85 ± 1.085d	22.18 ± 0.479bc
F2 (120 kg N ha^−1^ without yeast)		18.97 ± 0.704c	83.90 ± 2.279d	67.24 ± 0.759c	22.60 ± 0.512b
F3 (142 kg N ha^−1^ without yeast)		21.06 ± 0.537bc	106.37 ± 1.880bc	72.21 ± 0.945b	19.80 ± 0.313d
F4 (95 kg N ha^−1^ with yeast)		22.19 ± 0.810b	103.23 ± 4.497c	67.17 ± 0.912c	21.57 ± 0.383c
F5 (120 kg N ha^−1^ with yeast)		23.19 ± 0.597ab	117.44 ± 5.255b	67.72 ± 1.533c	19.92 ± 0.550d
F6 (142 kg N ha^−1^ with yeast)		25.27 ± 0.633a	129.41 ± 3.330a	75.22 ± 1.229a	19.54 ± 0.247d
**Interaction**					
G1	F0	–	–	42.11 ± 0.848g	30.53 ± 0.580a
	F1	17.47 ± 1.159f	75.51 ± 5.857j	64.27 ± 0.805ef	23.19 ± 0.421cd
	F2	21.18 ± 0.506bcdef	88.30 ± 3.399ghij	67.88 ± 1.650cdef	24.01 ± 0.367c
	F3	20.00 ± 0.494def	101.33 ± 2.121defghi	70.92 ± 1.300abcd	19.77 ± 0.846fg
	F4	22.28 ± 1.367abcde	99.96 ± 8.079efghi	66.81 ± 1.772def	22.37 ± 0.538cde
	F5	21.21 ± 0.616bcdef	102.45 ± 7.315defghi	68.93 ± 2.589bcdef	20.84 ± 1.084defg
	F6	23.94 ± 1.380abcd	123.54 ± 7.691abcd	74.07 ± 2.063abc	19.40 ± 0.523g
G2	F0	–	–	41.66 ± 1.175g	29.05 ± 1.535ab
	F1	19.20 ± 0.842def	90.67 ± 0.938fghij	63.69 ± 1.110f.	22.07 ± 1.027cdef
	F2	17.98 ± 0.770ef	79.43 ± 4.062ij	68.02 ± 0.269cdef	22.66 ± 0.379cde
	F3	22.86 ± 0.683abcd	112.29 ± 1.790bcdef	75.10 ± 0.780ab	20.35 ± 0.291efg
	F4	23.68 ± 1.844abcd	110.21 ± 1.446bcdefg	68.60 ± 0.926cdef	21.60 ± 0.556cdefg
	F5	23.56 ± 0.646abcd	120.45 ± 4.005abcde	66.90 ± 0.708def	19.57 ± 0.332fg
	F6	25.12 ± 0.691ab	127.42 ± 4.467abc	76.37 ± 1.102a	19.73 ± 0.227fg
G3	F0	–	–	41.44 ± 0.838g	27.98 ± 1.125b
	F1	20.49 ± 2.077cdef	95.27 ± 5.434fghij	63.60 ± 1.727f	21.27 ± 0.774defg
	F2	17.76 ± 1.202ef	83.98 ± 3.824hij	65.83 ± 1.619def	21.13 ± 0.895defg
	F3	20.32 ± 0.552cdef	105.47 ± 2.016cdefgh	70.61 ± 1.378abcde	19.27 ± 0.285g
	F4	20.60 ± 0.460bcdef	99.53 ± 3.142efghi	66.10 ± 2.166def	20.73 ± 0.701defg
	F5	24.82 ± 0.367abc	129.44 ± 8.538 ab	67.33 ± 2.376def	19.34 ± 1.303g
	F6	26.74 ± 0.683a	137.27 ± 1.967a	75.22 ± 2.441ab	19.49 ± 0.619g
**ANOVA**	**df**				
Genotypes (G)	2	< 0.001			
Fertilizer (F) level	6	< 0.001			
G × F	12	< 0.001			

### Seed yield response index (SYRI) of Canola

**Figure 4:**
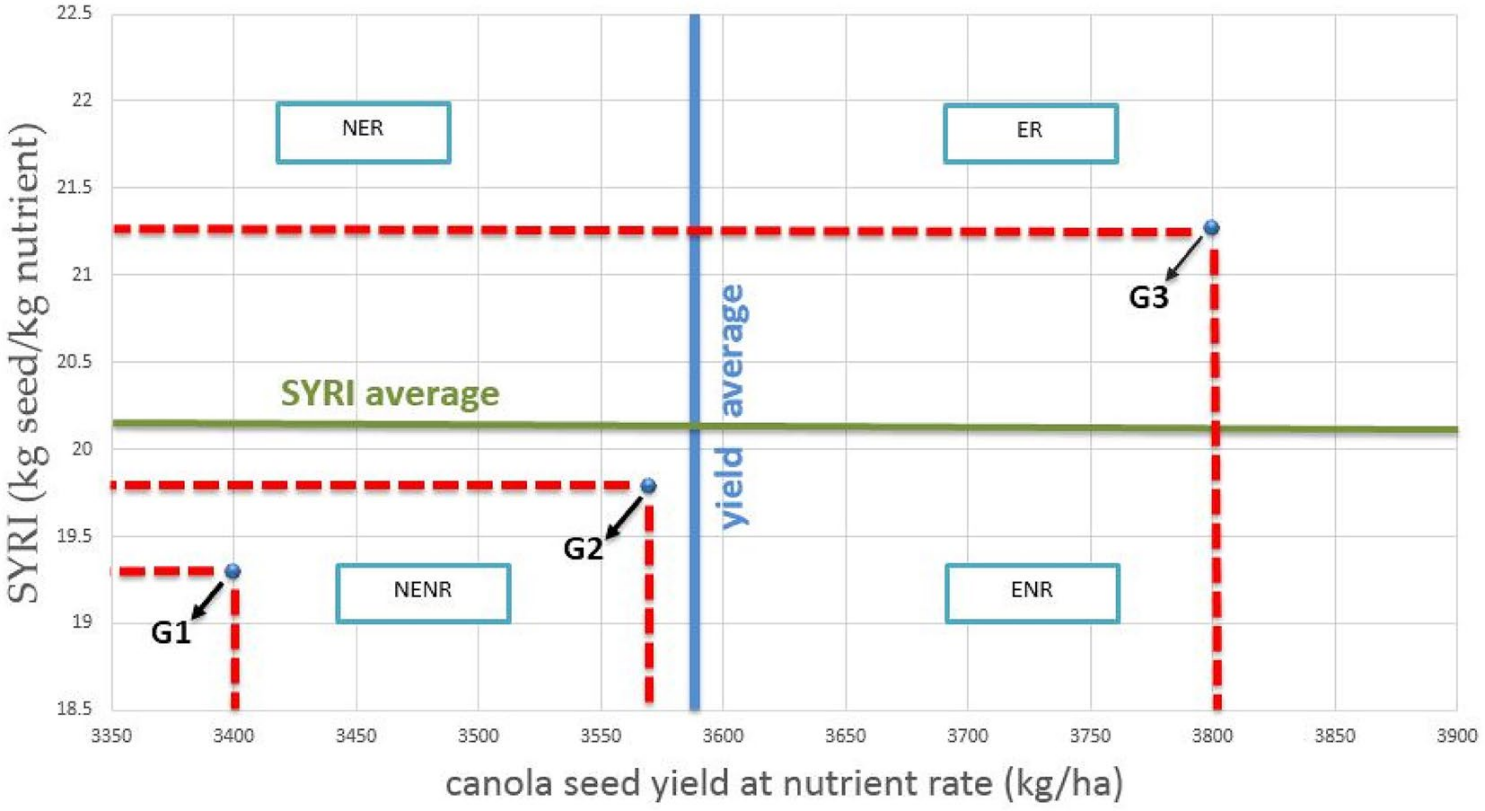
Seed yield response index (SYRI) of the tested Canola genotypes fertilized by nitrogen at a rate of 95 kg N ha^−1^, 120 kg N ha^−1^, 142 kg N ha^−1^, 95 kg N ha^−1^, + yeast extract, 120 kg N ha^−1^, + yeast extract, 142 kg N ha^−1^, + yeast extract. *ER* efficient and responsive, *NENR* neither efficient nor responsive.

Based on 0 kg N h^−1^ (control), as a low nutrient rate, and 142 kg N h^−1^ + yeast extract, as a high nutrient rate of nitrogen, SYRI of Canola was computed. SYRI pointed out the efficient genotype for producing higher seed yield at the low nutrient rate and their response to increasing nutrient fertilizer rates. In this connection, Fig. 4 illustrated that the average Canola seed yield at a low nutrient rate was 3590 kg h^−1^ as well as the mean SYRI value for 142 kg N h^−1^ + yeast extract was 20.12 kg seeds kg nutrient h^−1^. Accordingly, SemuDMK genotype was belonging to efficient and responsive (ER), being exceeded the averages of seed yield at the low nutrient rate and SYRI, while AD 201 and Topaz were neither efficient nonresponsive (NENR) since the seed yield at the low nutrient rate and SYRI were lower than the averages.

## Conclusion

Productivity and total seed N accumulation differed under different N and yeast extract management practices and canola cultivars. Results revealed that increasing rates of Nitrogen fertilization from 95 to 142 kg N ha^−1^ have a great effect on physico-chemical properties yield and its components. The result proved that 142 kg N ha^−1^ with yeast treatment was the best treatment for three canola genotypes. Regarding seed yield response index (SYRI) of canola, data cleared that SemuDMK genotype was belonging to efficient and responsive (ER), being exceeded the averages of seed yield at the low nutrient rate and SYRI, while AD 201 and Topaz were neither efficient nor nonresponsive (NENR) since the seed yield at the low nutrient rate and SYRI were lower than the averages.

## Materials and methods

### Plant material

The experimental research on plants, including the collection of plant material, complied with the relevant institutional, national, and international guidelines and legislation. Three Canola genotypes AD201 (G1), Topaz (G2), and SemuDNK 234/84 (G3) were used in this experiment, the first and third genotypes are Germany and the second is French. Canola genotypes seeds were secured from the Agricultural Research center (ARC), Ministry of Agriculture, Giza. Egypt.

### Experimental site

Two field experiments were carried out at the Experimental Station of the National Research Center, Nobaryia, Behaira Governorate, Egypt, during the winter seasons 2019–2020 and 2020–2021. Mechanical and chemical analysis of the soil experimental site is presented in Table 7 according to Chapman et al.^51^.

**Table 7:** Mechanical and chemical analysis of the experimental site soil.

Sand		Silt 20-0µ%	Clay < 2µ%	Soil texture							
Course 2000-200µ%	Fine 200-20µ %	12.66	4.18	Sandy							
47.64	36.59										

pH 1:2.5	EC dSm^−1^	CaCO_3_	OM%	Soluble Cations meq/l				Soluble anions meq/l			
				Na^+^	K^+^	Mg^+^	Ca^++^	CO_3_^–^	HCO^3−^	Cl^−^	SO_4_^–^
7.50	0.13	5.3	0.06	0.59	0.14	0.95	1.0	0.0	1.27	0.46	0.87

Available nutrients											
Macro element (ppm)			Micro element (ppm)								
N^−3^	P^−3^	K^+^		Zn^+2^	Fe^+2^	Mn^+2^	Cu^+2^				
51	12.2	74		0.13	1.3	0.28	0.00				

### Experimental treatments

Treatments were conducted as follows: without nitrogen fertilization (control F0), 95 kg N ha^−1^ (F1), 120 kg N ha^−1^ (F2) and 142 kg N ha^−1^ (F3) (without yeast); and integrated between nitrogen fertilization and yeast extract (YE) treatments as follows: 95 kg N ha^−1^ + YE (F4), 120 kg N ha^−1^ + YE (F5) and 142 kg N ha^−1^ + YE (F6) (with yeast). Nitrogen Fertilization treatments were applied as ammonium nitrate (33% N); it was added in equal twelve portions; the first dose was applied at sowing preparation, and the second was after 15 days from sowing, and the other portions were added weekly with irrigation water requirements. Yeast extract was added as (60 L ha^−1^) throughout the irrigation system in the same order as nitrogen fertilization. Plants received the recommended doses of Phosphorus and potassium fertilizers before sowing at the rate of (475) and (120) kg ha^−1^ of calcium supper phosphate (15.5% P_2_O_5_) and potassium sulphate (48% K_2_O), respectively.

### Experimental design

The trial design was carried out as a strip plot design with three replications; Fertilizer treatments occupied the vertical main plots and the cultivars were distributed in horizontal ones. The experimental unit area was 16.8 m consisting of 6 ridges 4 m in length and 0.70 m in width and planted one ridge side at 15 cm apart and one plant per hill. The drip irrigation system was installed; the drip lateral had emitters spaced 20 cm apart with a nominal discharge of 4 L h^−1^.

### Yeast extract preparation

Yeast (*Saccharomyces cerevisiae*) extract was prepared by using a technique that allowed yeast cells (pure active dry yeast 100 g L^−1^) to be grown and multiplied efficiently during conducive aerobic and nutritional conditions that allowed to produce beneficial bio-constituent i.e. carbohydrates, sugars, proteins, amino acids, fatty acids, hormones, etc^52^. The chemical analysis of yeast extract was analyzed by Ref.^35^ as presented in Table 8.

**Table 8:** Chemical analysis of yeast extract.

Amino acid		Vitamins and carbohydrates	
(mg 100–1 g dry weight)			
Arginine	1.99	Vitamin B1	2.23
Histidine	2.63	Vitamin B2	1.33
Isoleucine	2.31	Vitamin B6	1.25
Leucine	3.09	Vitamin B12	0.15
Lysine	2.95	Thimain	2.71
Methionine	0.72	Riboflavin	4.96
Phenyl alanine	2.01	Insitol	0.26
Threonine	2.09	Biotin	0.09
Tryptophan	0.45	Nicotinic acid	39.88
Valine	2.19	Panthothenic acid	19.56
Glutamic acid	2.00	P amino benzoic acid	9.23
Serine	1.59	Folic acid	4.36
Aspartic acid	1.33	Pyridoxine	2.90
Cysteine	0.23	Total carbohydrates	23.2
Proline	1.53		
Tyrosine	1.49	Glucose	13.33

### Sampling and collecting data

Samples of guarded Canola plants were randomized and collected in both seasons; the first sample was taken 90 days after sowing, wherein ten plants were collected from each treatment to determine Chlorophyll a and b calorimetrically in fresh leaves (mg g^−1^ FW) according to the methods described by Ref.^53^ then calculated Chlorophyll a/b ratio; the second sample ten plants were taken at the harvesting time (180 days after sowing) to measured plant heights (cm), number of branches, pods number/plant and number of seed/pods; All plants (140 plants) in each plot (16.8 m) were harvested to determine the seed yield (kg ha^−1^), and biological yield (kg ha^−1^) and Harvest index.

### Chemical composition of Canola genotypes

The chemical composition of samples was determined i.e. oil yield, seed oil %, protein %, N % in seed, and N % in straw. Oil % of seed was determined by solvent extraction method according to Dolatabadian et al.^54^. Protein content in seeds was calculated by multiplying N content by 6.25. Determination of Physico-chemical properties i.e. Acid value (mg g^−1^), Saponification number (mg g^−1^) and peroxide value (mg kg^−1^) were determined as the method described by Dolatabadian et al.^54^.

Crude oil of seeds (2nd season only) was used as authentic material for identification of the following fatty acids according to Stahl et al.^55^ The amount of each fatty acid in the oil under investigation was determined according to Nelson et al.^56^. Note: The statistical analysis does not do on some parameters such as fatty acids composition.

### Nitrogen efficiency indexes

Nitrogen efficiency indexes were calculated as follows: Nitrogen use efficiency, NUE = seed yield kg h^−1^/applied nitrogen kg h^−1^; Nitrogen remobilization efficiency (NRE) = total N uptake kg h^−1^ × 100/N applied kg h^−1^; Nitrogen harvest index (NHI) total N in seeds kg h^−1^ × 100/total N uptake kg h^−1^, and nitrogen physiological efficiency (NPE, seed yield (kg h^−1^)/total N uptake kg h^−1^) were calculated according to Timsina et al.^57^.

### Seed yield response index

Seed yield response index (SYRI) was calculated for each genotype using formula of Fageria and Barbosa Filho^58^ as follow: SYRI (kg seeds kg nutrient − 1) = (SY at high nutrient rate − SY at low nutrient rate)/(high nutrient rate − low nutrient rate). Where SY: seed yield kg h^−1^, Low nutrient rate = 95 kg N h^−1^, High nutrient rate = 142 kg N h^−1^ + yeast extract.

According to the SYRI value, genotypes could be classified into four groups: (i) efficient and responsive (ER) that produce high seed yield at low as well as high rates of nutrient fertilizer; (ii) efficient and not responsive (ENR) that produce high seed yield at low nutrient rate with lower response to increase nutrient fertilizer than ER; (iii) not efficient but responsive (NER) that has low seed yield with response to increase nutrient fertilizer; and (iv) neither efficient nor responsive (NENR) that has low seed yield with low response to increase nutrient fertilizer.

### Statistical analysis

The obtained data were exposed to the proper statistical analysis according to Snedecor et al.^59^. Using Costat computer program V 6.303 (2004). Duncan Multiple Range Test^60^ in the probability level of 5% level of significance was used to differentiate between means, and Correlation coefficient according to Afiah et al.^61^. Data of both growing seasons were subjected to a homogeneity variance test for running the combined analysis of the data.

## Data Availability

All the data of the current work are included in the submitted article. For further data, please contact the corresponding author (M.E.) via mohamedebaid979@gmail.com.

## References

[1] US Department of Agriculture, A. R. S. USDA national nutrient database for standard reference, release 28. *Nutrient data laboratory home page* (2011).

[2] Ghallab K, Sharaan A (2002). Selection in canola (*Brassica napus* L.) germplasm under conditions of newly reclaimed land. II. Salt tolerant selections. Egypt. J. Plant Breed..

[3] Yasari, E., Azadgoleh, A. E., Pirdashti, H. & Mozafari, S. Azotobacter and Azospirillum inoculants as biofertilizers in canola (Brassica napus L.) cultivation. *Asian J. Plant Sci.* (2008).

[4] Brown, J., Davis, J., Lauver, M. & Wysocki, D. USCA Canola Growers’ Manual. *Oregon. P* **71** (2008).

[5] Kamel, S., Mahfouz, H., Blal, H., Said, M. & Mahmoud, M. Effects of salicylic acid elicitor and potassium fertilizer as foliar spray on canola production in the reclaimed land in Ismailia Governorate, Egypt. (2016).

[6] Din J, Khan S, Ali I, Gurmani A (2011). Physiological and agronomic response of canola varieties to drought stress. J. Anim. Plant Sci..

[7] Massignam A, Chapman S, Hammer G, Fukai S (2009). Physiological determinants of maize and sunflower grain yield as affected by nitrogen supply. Field Crop Res..

[8] Sultana SR, Ahmad A, Wajid A, Akhtar J (2013). Estimating growth and yield related traits of wheat genotypes under variable nitrogen application in semi-arid conditions. Pak. J. Life Soc. Sci..

[9] Patel J, Shelke V (1998). Effect of farmyard manure, phosphorus and sulphur on growth, yield and quality of Indian mustard (*Brassica juncea*). Indian J. Agron..

[10] Ahmadi M, Bahrani M (2009). Yield and yield components of rapeseed as influenced by water stress at different growth stages and nitrogen levels. Am. Eurasian J. Agric. Environ. Sci..

[11] Al-Solaimani SG, Alghabari F, Ihsan MZ (2015). Effect of different rates of nitrogen fertilizer on growth, seed yield, yield components and quality of canola (*Brassica napus* L.) under arid environment of Saudi Arabia. Int. J. Agron. Agric. Res..

[12] Sainju, U. M., Ghimire, R. & Pradhan, G. P. Nitrogen fertilization I: Impact on crop, soil, and environment. *Nitrogen Fixat.* (2019).

[13] Hafeez K, Zhang Y, Malak N (2002). Core competence for sustainable competitive advantage: A structured methodology for identifying core competence. IEEE Trans. Eng. Manag..

[14] Salantur A, Ozturk A, Akten S, Sahin F, Donmez F (2005). Effect of inoculation with non-indigenous and indigenous rhizobacteria of Erzurum (Turkey) origin on growth and yield of spring barley. Plant Soil.

[15] El-Ghamriny, E., Arisha, H. & Nour, K. Studies on tomato flowering, fruit set, yield and quality in summer season 1-spraying with Thiamine, ascorbic acid and yeast. *Zagazig J. Agric. Res. Egypt.* (1999).

[16] Wanas A (2002). Resonance of faba bean (*Vicia faba* L.) plants to seed soaking application with natural yeast and carrot extracts. Ann. Agric. Sci. Moshtohor.

[17] Glick BR (1995). The enhancement of plant growth by free-living bacteria. Can. J. Microbiol..

[18] Barnett, J. A., Payne, R. W. & Yarrow, D. Yeasts: characteristics and identification. (1990).

[19] Sarhan TZ, Mohammed GH, Teli JA (2011). Effect of bio and organic fertilizers on growth, yield and fruit quality of summer squash. Sarhad J. Agric..

[20] Feng X, An Y, Gao J, Wang L (2020). Photosynthetic responses of canola to exogenous application or endogenous overproduction of 5-aminolevulinic acid (ALA) under various nitrogen levels. Plants.

[21] Liu C (2020). Low-nitrogen tolerance comprehensive evaluation and physiological response to nitrogen stress in broomcorn millet (*Panicum miliaceum* L.) seedling. Plant Physiol. Biochem..

[22] Vouillot MO, Huet P, Boissard P (1998). Early detection of N deficiency in a wheat crop using physiological and radiometric methods. Agronomie.

[23] Cruz J, Mosquim P, Pelacani C, Araújo W, DaMatta F (2003). Photosynthesis impairment in cassava leaves in response to nitrogen deficiency. Plant Soil.

[24] Terashima I, Hikosaka K (1995). Comparative ecophysiology of leaf and canopy photosynthesis. Plant Cell Environ..

[25] Evans JR (1999). Leaf anatomy enables more equal access to light and CO_2_ between chloroplasts. New Phytol..

[26] Hikosaka K, Terashima I (1995). A model of the acclimation of photosynthesis in the leaves of C3 plants to sun and shade with respect to nitrogen use. Plant Cell Environ..

[27] Terashima I, Evans JR (1988). Effects of light and nitrogen nutrition on the organization of the photosynthetic apparatus in spinach. Plant Cell Physiol..

[28] Thompson W, Huang L, Kriedemann P (1992). Photosynthetic response to light and nutrients in sun-tolerant and shade-tolerant rainforest trees. II. Leaf gas exchange and component processes of photosynthesis. Funct. Plant Biol..

[29] Kitajima K, Hogan KP (2003). Increases of chlorophyll a/b ratios during acclimation of tropical woody seedlings to nitrogen limitation and high light. Plant Cell Environ..

[30] Bungard R, Scholes J, Press M (2000). The influence of nitrogen on rain forest dipterocarp seedlings exposed to a large increase in irradiance. Plant Cell Environ..

[31] Abd El-Motaleb H, Gomaa A (2004). Yield response of two canola varieties to nitrogen and biofertilizers under sandy soil conditions. Agric. Res. J. Suez Canal Univ..

[32] Takahashi CA, Mercier H (2011). Nitrogen metabolism in leaves of a tank epiphytic bromeliad: characterization of a spatial and functional division. J. Plant Physiol..

[33] Tawfiq AA (2010). Estimation levels of Indol acetic acid (IAA) and Gibberellic acid (GA3) from dry bakery yeast *Saccharomyces cereviciae*. J. Biotechnol. Res. Center.

[34] Wanas A (2007). trials for improving growth and productivity of tomato (*Lycopersicon esculentum*, Mill.) plants grown in winter season. J. Plant Prod..

[35] Mahmoued, T. *Botanical studies on the growth and germination of mahnolia (Magnolia grandiflora L.) plants. M. Sci*, Thesis. Fac. of Agric. Moshtohor, Zagazig University (2001).

[36] Hocking P, Stapper M (2001). Effects of sowing time and nitrogen fertiliser on canola and wheat, and nitrogen fertiliser on Indian mustard. I. Dry matter production, grain yield, and yield components. Aust. J. Agric. Res..

[37] Ahmad G, Jan A, Arif M, Jan M, Khattak R (2007). Influence of nitrogen and sulfur fertilization on quality of canola (*Brassica napus* L.) under rainfed conditions. J. Zhejiang Univ. Sci. B.

[38] Sharma D, Pathak D, Atwal A, Sangha M (2009). Genetic variation for some chemical and biochemical characteristics in cotton seed oil. J. Cotton Res. Dev..

[39] Cornelius J (1966). Some technical aspects influencing the quality of palm kernels. J. Sci. Food Agric..

[40] Choe E, Min DB (2006). Mechanisms and factors for edible oil oxidation. Compr. Rev. Food Sci. Food Saf..

[41] Gunstone, F. D. *Rapeseed and Canola Oil: Production, Processing, Properties and Uses* (CRC Press, 2004).

[42] El Kholy, M., El-Zeky, M., Saleh, S. Z. & Metwaly, S. In *Proc. Proceedings of the 12th International Rapeseed Congress (2007).* 26–30.

[43] El Habbasha, E. S. F. & El Salam, M. A. Response of two canola varieties (*Brassica napus* L.) to nitrogen fertilizer levels and zinc foliar application. (2009).

[44] Mekki, B. In *Proceedings 11th International Rapeseed Congress, Copenhagen.* 915–917.

[45] Mekki B (2013). Yield and quality traits of some canola varieties grown in newly reclaimed sandy soils in Egypt. World Appl. Sci. J..

[46] Rahman M (2002). Fatty acid composition of resynthesized *Brassica napus* and trigenomic Brassica void of genes for erucic acid in their A genomes. Plant Breed..

[47] El-Beltagi HE-DS, Mohamed AA (2010). Variations in fatty acid composition, glucosinolate profile and some phytochemical contents in selected oil seed rape (*Brassica napus* L.) cultivars. Grasas Aceites.

[48] Khan S (2017). Optimization of nitrogen rate and planting density for improving yield, nitrogen use efficiency, and lodging resistance in oilseed rape. Front. Plant Sci..

[49] Hirel B, Tétu T, Lea PJ, Dubois F (2011). Improving nitrogen use efficiency in crops for sustainable agriculture. Sustainability.

[50] Laufer D, Nielsen O, Wilting P, Koch H-J, Märländer B (2016). Yield and nitrogen use efficiency of fodder and sugar beet (*Beta vulgaris* L.) in contrasting environments of northwestern Europe. Eur. J. Agron..

[51] Chapman HD, Pratt PF (1962). Methods of analysis for soils, plants and waters. Soil Sci..

[52] Spencer, J. F., Spencer, D. M. & Smith, A. *Yeast Genetics: Fundamental and Applied Aspects*. (Springer Science & Business Media, 2012).

[53] Hiscox J, Israelstam G (1979). A method for the extraction of chlorophyll from leaf tissue without maceration. Can. J. Bot..

[54] Dolatabadian A, Sanavy SM, Chashmi N (2008). The effects of foliar application of ascorbic acid (vitamin C) on antioxidant enzymes activities, lipid peroxidation and proline accumulation of canola (*Brassica napus* L.) under conditions of salt stress. J. Agron. Crop Sci..

[55] Stahl WR (1967). Scaling of respiratory variables in mammals. J. Appl. Physiol..

[56] Nelson J, Milun A, Fisher H (1970). Gas chromatographic determination of tocopherols and sterols in soya sludges and residues—An improved method. J. Am. Oil. Chem. Soc..

[57] Timsina J, Singh U, Badaruddin M, Meisner C, Amin M (2001). Cultivar, nitrogen, and water effects on productivity, and nitrogen-use efficiency and balance for rice–wheat sequences of Bangladesh. Field Crop Res..

[58] Fageria, N. & Barbosa Filho, M. Screening rice cultivars for higher efficiency of phosphorus absorption [*Oryza sativa*]. *Pesquisa Agropecuaria Brasileira* (1981).

[59] Snedecor, G. & Cochran, W. *Statistical Methods*, 6th ed. 507 (Iowa State Univ Press, 1990).

[60] Duncan DB (1955). Multiple range and multiple F tests. Biometrics.

[61] Afiah S, Ghoneim E (2000). Correlation, stepwise and path coefficient analysis in Egyptian cotton under saline conditions. Arab. Univ. J. Agric. Sci.

